# Ex Vivo Chromosomal Radiosensitivity Testing in Patients with Pathological Germline Variants in Breast Cancer High-Susceptibility Genes *BReast CAncer 1* and *BReast CAncer 2*

**DOI:** 10.3390/cimb45080418

**Published:** 2023-08-10

**Authors:** Tara Zuhair Kassem, Marius Wunderle, Lukas Kuhlmann, Matthias Ruebner, Hanna Huebner, Juliane Hoyer, André Reis, Peter A. Fasching, Matthias W. Beckmann, Carolin C. Hack, Rainer Fietkau, Luitpold Distel

**Affiliations:** 1 Department of Radiation Oncology, Universitätsklinikum Erlangen, Friedrich-Alexander-Universität Erlangen-Nürnberg, Universitätsstraße 27, D-91054 Erlangen, Germany; tarazuhairkassem@yahoo.de (T.Z.K.); lukas.kuhlmann@uk-erlangen.de (L.K.); rainer.fietkau@uk-erlangen.de (R.F.); 2 Department of Gynecology and Obstetrics, Universitätsklinikum Erlangen, Friedrich-Alexander-Universität Erlangen-Nürnberg, Universitätsstraße 27, D-91054 Erlangen, Germany; marius.wunderle@t-online.de (M.W.); matthias.ruebner@uk-erlangen.de (M.R.); hanna.huebner@uk-erlangen.de (H.H.); peter.fasching@uk-erlangen.de (P.A.F.); matthias.beckmann@uk-erlangen.de (M.W.B.); carolin.hack@uk-erlangen.de (C.C.H.); 3 Institute of Human Genetics, Universitätsklinikum Erlangen, Friedrich-Alexander-Universität Erlangen-Nürnberg, Schwabachanlage 10, D-91054 Erlangen, Germany; juliane.hoyer@uk-erlangen.de (J.H.); andre.reis@uk-erlangen.de (A.R.); 4 Centre for Rare Diseases Erlangen (ZSEER), Universitätsklinikum Erlangen, D-91054 Erlangen, Germany

**Keywords:** *BRCA1*, *BRCA2*, breast cancer, chromosomal radiosensitivity, FISH assay, radiation oncology, radiotherapy, radiation sensitivity testing

## Abstract

Background: Individual radiosensitivity is an important factor in the occurrence of undesirable consequences of radiotherapy. The potential for increased radiosensitivity has been linked to highly penetrant heterozygous mutations in DNA repair genes such as *BRCA1* and *BRCA2*. By studying the chromosomal radiosensitivity of *BRCA1/2* mutation carriers compared to the general population, we study whether increased chromosomal radiation sensitivity is observed in patients with *BRCA1/2* variants. Methods: Three-color-fluorescence in situ hybridization was performed on ex vivo-irradiated peripheral blood lymphocytes from 64 female patients with a heterozygous germline *BRCA1* or *BRCA2* mutation. Aberrations in chromosomes #1, #2 and #4 were analyzed. Mean breaks per metaphase (B/M) served as the parameter for chromosomal radiosensitivity. The results were compared with chromosomal radiosensitivity in a cohort of generally healthy individuals and patients with rectal cancer or breast cancer. Results: Patients with *BRCA1/2* mutations (*n* = 64; B/M 0.47) overall showed a significantly higher chromosomal radiosensitivity than general healthy individuals (*n* = 211; B/M 0.41) and patients with rectal cancer (*n* = 379; B/M 0.44) and breast cancer (*n* = 147; B/M 0.45) without proven germline mutations. Chromosomal radiosensitivity varied depending on the locus of the *BRCA1/2* mutation. Conclusions: *BRCA1/2* mutations result in slightly increased chromosomal sensitivity to radiation. A few individual patients have a marked increase in radiation sensitivity. Therefore, these patients are at a higher risk for adverse therapeutic consequences.

## 1. Introduction

Breast cancer is the most common cancer worldwide, and the leading cause of cancer death in women [1,2]. Every eighth woman in North America and Northern/Western Europe develops breast cancer during her lifetime [3]. Approximately 15–20% have a familial background [4,5] and 5–10% occur due to mutations in high (e.g., *BRCA1*, *BRCA2*, *CDH1*, *PALB2*, *PTEN*, *STK11*, *TP53*)- or moderate (e.g., *ATM*, *BARD1*, *CHEK2*, *RAD51C/D*)-susceptibility genes [4,6]. Mutations in *BRCA1/2* are most common, causing a significantly increased risk of breast cancer of about 50–70% (*BRCA1)* and 40–60% (*BRCA2*) [4,6,7,8] as well as of ovarian cancer of about 40–50% (*BRCA1)* and 15–25% (*BRCA2)* [7,8] by 70–80 years of age. *BRCA1/2* are also associated with an elevated risk of male breast cancer, prostate cancer, pancreatic cancer and other entities [9]. Among the genetic variants that are associated with an increased susceptibility to tumors, there is also a fraction that is associated with an increased sensitivity to radiation [10].

*BRCA1* located on chromosome 17q21 and *BRCA2* located on chromosome 13q12.3 encode essential DNA damage repair proteins that interact with a variety of proteins responsible for genome stability and cell cycle control [11]. They are involved in both the detection and repair of double-strand breaks. The indirect effects on the repair of single-strand breaks and of complex single strands through poly-ADP-ribose polymerase (PARP) are successfully exploited by the use of PARP-inhibitors in different *BRCA1/2*-mutated cancer entities [11,12]. Genomic alterations in these genes lead to specific molecular types of breast cancer and have implications for therapy planning [12].

According to international guidelines, radiotherapy is the standard of care in all primary breast cancer patients after breast-conserving therapy and also in those after mastectomy with risk factors to reduce the risk of recurrence and death. The German AWMF association recommends conventional irradiation with a total dose between 45 and 50 Gy with single doses of 1.8–2 Gy per session or hypofractionation with a total dose of 40 Gy in 15–16 fractions [13].

Increased radiosensitivity is among factors that are associated with a higher rate of adverse effects from irradiation [10], which can impact quality of life and can be potentially fatal [14,15,16,17,18]. In these cases, monitoring and intensified follow-up are recommended. Thus, above-average-sensitive patients should be identified [19].

The aim of this study was to determine whether pathogenic heterozygous germline variants in the *BRCA1/2* genes confer increased chromosomal radiosensitivity compared with measurements of chromosomal radiosensitivity in a cohort of generally healthy individuals, patients with rectal cancer and patients with breast cancer. In addition, we were interested in whether specific *BRCA1/2* mutations may be associated with increased chromosomal radiosensitivity.

To assess the individual chromosomal radiosensitivity, a three-color-fluorescence in situ hybridization (FiSH) assay was performed that permits an ex vivo analysis of chromosomal radiosensitivity. In the assay, chromosomal aberrations resulting from 2 Gy ex vivo irradiation with ionizing radiation are counted as breaks per metaphase (B/M) and compared to control groups. We considered B/M above 0.5 as increased chromosomal radiation sensitivity and assumed that it is equal to individual radiation sensitivity. We recommend intensified follow-up care from a B/M above 0.55.

## 2. Material and Methods

### 2.1. Patient Recruitment

Venous blood samples of 64 patients with a germline mutation in *BRCA1* or *BRCA2* were drawn for a three-color FiSH assay. Patients were either consecutively sampled at the department of gynecology and obstetrics of the university hospital of Erlangen-Nürnberg (*n* = 59), or radiosensitivity testing was requested by various clinics in Germany because of a *BRCA1/2* mutation (*n* = 5). A total of 38 of the *BRCA*+ patients (*BRCA1*, *n* = 26, mean age = 48.8 years, *BRCA2*, n = 12/51.0) had been diagnosed with breast cancer. There were 26 patients who were identified as *BRCA*+ (*BRCA1*, *n* = 11/37.5, *BRCA2*, n = 15/38.8) based on their family history, but who did not have an oncogenic disease. Written informed consent was collected from all included patients. Only one sample could not be considered due to low numbers of metaphases. This study was approved by the ethics review committee of the Friedrich-Alexander University Erlangen-Nürnberg (21_19 B). Patients who underwent radiotherapy within the three previous months were excluded. The control cohorts were consecutively sampled at the radiotherapy department of the university hospital of Erlangen-Nürnberg. These include data from healthy individuals (*n* = 211, mean age 50.1, range 18–81; 121 female, 90 male), rectal cancer patients (*n* = 379, mean age 57.3, range 28–91; 101 female, 278 male) and breast cancer patients (*n* = 147), which have been published previously [20,21,22]. The control cohorts were selected to have a healthy cohort with few likely pathogenic genetic variants. The comparison cohort of rectal cancer patients was chosen as a cancer condition mainly induced by noxious agents, while a cohort with breast cancer patients was selected for comparison, in which patients with genetic variants were expected to be more prevalent. In the entire control cohort, the mutation status of *BRCA1/2* or other mutations associated with increased chromosomal sensitivity to radiation was not known.

### 2.2. Chromosome Preparation and the Three-Color Fluorescence In Situ Hybridisation

At least 8 mL of venous blood was drawn in heparin tubes (NH4-Heparin, Sarstedt, Nürnbrecht, Germany). Half of the sample was irradiated ex vivo with 2 Gy in a tissue block by a 6 MV linear accelerator (Mevatron, Siemens, Germany). A dose of 2 Gy corresponds to a fractioned dose per day patients receive during radiotherapy [23]. The other half of the specimen served as a control and was not irradiated. In a culture medium of RPMI, 2.5% phytohemagglutinin, 1% penicillin/streptomycin and 15% fetal calf serum, both portions were incubated for 48 h at a temperature of 37 °C [20]. The peripheral lymphocytes were stimulated by phytohemagglutinin [24]. Lymphocytes were arrested with 0.1 µg/mL of colcemid (Gibco, Wlatham, MS, USA) in the metaphase of the first cell division. After 3.5 h, the culture slides were prepared for isolation of human lymphocytes. Potassium chloride was used to swell the chromosomes. Cultures were fixed by methanol and acetic acid. A clear platelet remained from which the cell suspension was dropped onto slides. Slides were then further prepared.

DNA was hybridized to be able to stain chromosomes #1, #2 and #4. For their staining, fluorescence dyes (Bio-FITC/Dig-Rhodamin; Molecular Probes, Karlsruhe, Germany) were used in the colors red, yellow and green. At the end of the procedure, the chromosomes were counterstained blue with DAPI (Molecular Probes, Karlsruhe, Germany) and covered with Vectashield (Newark, CA, USA) [25,26]. Specimens were visualized by fluorescence microscopy (Zeiss, Axioplan 2, Göttingen, Germany). At least 200 metaphases each of unirradiated blood and irradiated blood were evaluated for chromosomal aberrations and the background was subtracted from those irradiated.

### 2.3. Image Analysis

Stained chromosomes in the metaphase stage were imaged on a fluorescence microscope (Zeiss, Axioplan 2, Göttingen, Germany) at 630x magnification. The Metasystems software (Metapher 4 V3.10.1, Altlussheim, Germany) was used. First, an automatic detection of chromosomes was performed at 100x amplification. Further, a specified capture of each metaphase in different colors was conducted using the microscope at a magnification of 630x.

The colored images were used for further analyses of chromosomal breaks. An image evaluation software (Biomas 6.1, Erlangen, Germany) served as an input mask to evaluate each metaphase manually. Results were automatically transferred to an Excel spreadsheet (Excel, Microsoft Corporation, Redmond, WA, USA) by Biomas. At least 200 metaphases were assessed each [26].

Translocations, dicentric chromosomes, acentric chromosomes, rings, deletions, insertions and complex chromosomal rearrangements were detected and evaluated in the three stained chromosomes [25,27]. The aberrations were scored by the number of underlying chromosomal breaks according to Savage and Simpson [28]. Therefore, breaks and deletions were counted as one break; translocations, dicentric and rings as two breaks; and insertions as three breaks. Complex aberrations were scored according to how many breaks were theoretically necessary for their formation. Thus, all chromosomal aberrations were considered and summarized in the value breaks per metaphase (B/M). Scores (B/M) were successively projected into an Excel spreadsheet. The B/M value of the irradiated sample was corrected by the B/M value of the non-irradiated control sample [29].

### 2.4. Statistical Analysis

For statistical analysis, we applied SPSS Statistics 22.0 (IBM, Armonk, NY, USA) [30,31]. Levene’s test and the two-sided T-test were used to test for significant differences between both groups, Pearson’s r correlation was calculated to test possible correlations and Fisher’s exact test to compare the distribution of different groups of radiosensitivity. *p* values < 0.05 were regarded as significant [23]. For visualizing our data and statistical analysis, GraphPad Prism (2020) was utilized.

## 3. Results

### 3.1. Patient Characteristics

The study cohort consisted of blood samples from 37 female patients with a pathogenic heterozygous germline mutation in *BRCA1* and 27 female patients with a mutation in *BRCA2* according to ACMG criteria. The radiosensitivity of female *BRCA1/2* mutation carriers (*n* = 64, mean age 45.7 years) was compared with that of male and female healthy subjects (*n* = 211, mean age 50.3 years). In addition, it was compared with that of rectal cancer patients (*n* = 379, mean age 63.1 years) and breast cancer patients (*n* = 147, mean age 57.3 years) without a known mutation status. Of the 64 patients with a pathological variant of *BRCA1/2*, 38 patients were diagnosed with breast cancer. In the remaining individuals, *BRCA1/2* status was determined based on family history. The majority of these breast cancers were in an early stage T1 or T2, with a high proportion of triple-negative tumors in both *BRCA1/2*-mutated groups (Table 1).

### 3.2. Chromosomal Radiosensitivity Testing

**Figure 1:**
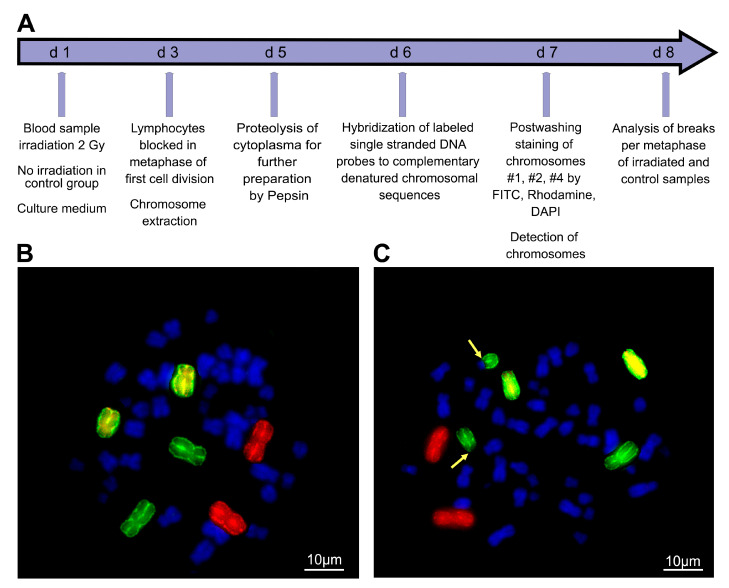
Chromosomal radiosensitivity testing procedure and analysis of chromosomal aberrations scored as breaks per metaphase. (**A**) Chromosomal radiation sensitivity test procedure of ex vivo blood for irradiation and generation of chromosomal aberrations. (**B**) Unaffected metaphase with red stained chromosome #1, green chromosome #2 and yellow chromosome #4. (**C**) Chromosome #2 involved in a translocation depicted by yellow arrows.

Chromosomal radiation sensitivity of blood lymphocytes was studied using three-color G0 FiSH. Aberrations in chromosomes #1, #2 and #4 induced by ionizing radiation were analyzed for this purpose [26]. Background aberrations and aberrations after irradiation with 2 Gy ionizing radiation (IR) were analyzed and the background was subtracted from the IR-induced aberrations (Figure 1).

**Figure 2:**
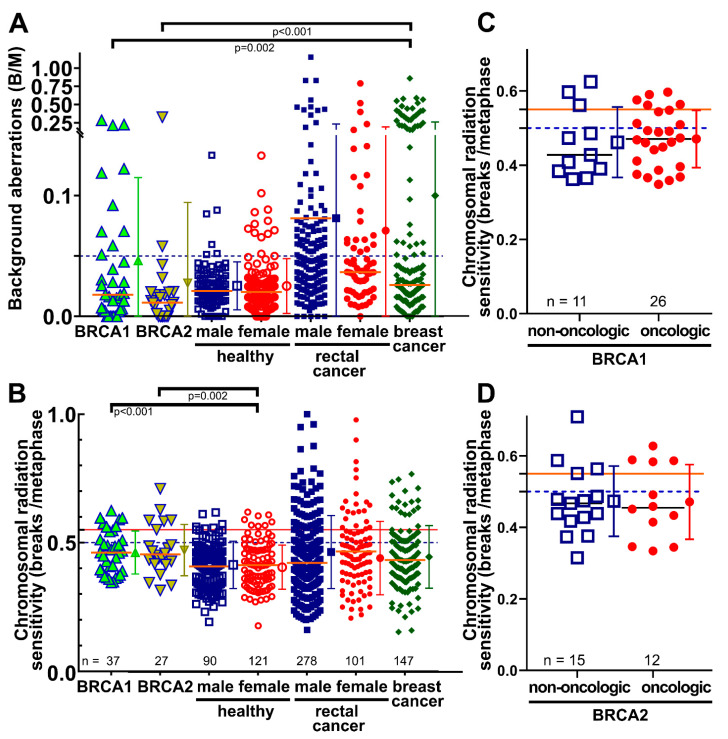
Chromosomal aberrations scored as breaks per metaphase in different cohorts. The entire cohort consisted of 37 female patients with pathogenic mutations in *BRCA1* and 27 patients with pathogenic mutations in *BRCA2*, 121 female and 90 male healthy individuals, 101 female and 278 male patients with rectal cancer, and 147 female patients with breast cancer (the latter ones all with unknown *BRCA1* mutation status). (**A**) Background aberrations for breaks per metaphase and (**B**) chromosomal radiation sensitivity scored as breaks per metaphase after ex vivo irradiation with 2 Gy ionizing radiation and background correction. (**C**) B/M values in the non-oncologic and oncologic *BRCA1* group. (**D**) B/M values in the non-oncologic and oncologic *BRCA2* group. Brackets indicate significant levels of differences between two groups. Horizontal lines depict 0.05 (**A**), and 0.5 and 0.55 (**B**) B/M cut-off values. B/M = Breaks per metaphase. Error bars indicate the mean value and the standard deviation.

Background aberrations in the cohort with a *BRCA1* pathologic variant (0.047 B/M ± standard deviation 0.069) and a *BRCA2* pathologic variant (0.012 B/M ± 0.013) were overall comparable as in the control group of healthy female individuals with unknown *BRCA1/2* mutation status (0.025 B/M ± 0.023) (*p* = 0.072 and 0.863). Although there was no difference between *BRCA1* and *BRCA2* (*p* = 0.290), the *BRCA1*-mutated cohort had a markedly higher proportion of 27.8% with elevated background levels above 0.05 B/M (Table 2). This was similar to the group of women with rectal cancer (25.2%) or with breast cancer (30.6%) with unknown mutation status (Figure 2A).

### 3.3. B/M Values in Patient Cohorts

After ex vivo irradiation and background subtraction, the chromosomal radiation sensitivity of patients with *BRCA1* mutations (0.464 B/M ± 0.083) or *BRCA2* mutations (0.476 B/M ± 0.099) was quite similar (*p* = 0.706) (Table 3). Overall, patients with *BRCA1* and *BRCA2* pathologic variants had higher mean B/M values compared to the healthy control cohorts (Figure 2B). The *BRCA1* cohort and *BRCA2* cohort were clearly more chromosomal-radiosensitive than the female (*n* = 121) healthy cohort (*p* < 0.001 and *p* = 0.002) and the total healthy cohort (*p* < 0.001 and *p* = 0.002). Elevated chromosomal radiosensitivity above the threshold of 0.5 B/M was found in 18 patients, nine above 0.55 and three above 0.6 B/M (Table 2). There was no difference in B/M values between men and women in the healthy group (*p* = 0.954).

There was no difference between the *BRCA1* cohort and the *BRCA2* cohort and the female cohort with advanced rectal cancer (*p* = 0.359 and *p* = 0.187) as well as with the total cohort of advanced rectal cancer (*p* = 0.190 and *p* = 0.050). Breast cancer patients were not different from *BRCA1-* (*p* = 0.405) or *BRCA2-* (*p* = 0.191) mutated patients (Table 3) (Figure 2B). Similarly, healthy individuals and cancer patients with either *BRCA1* (*p* = 0.700) or *BRCA2* (*p* = 0.999) did not differ from each other (Figure 2C,D).

### 3.4. Radiation Sensitivity among the Non-Oncologic and Oncologic BRCA1/2-Mutated Individuals

**Figure 3:**
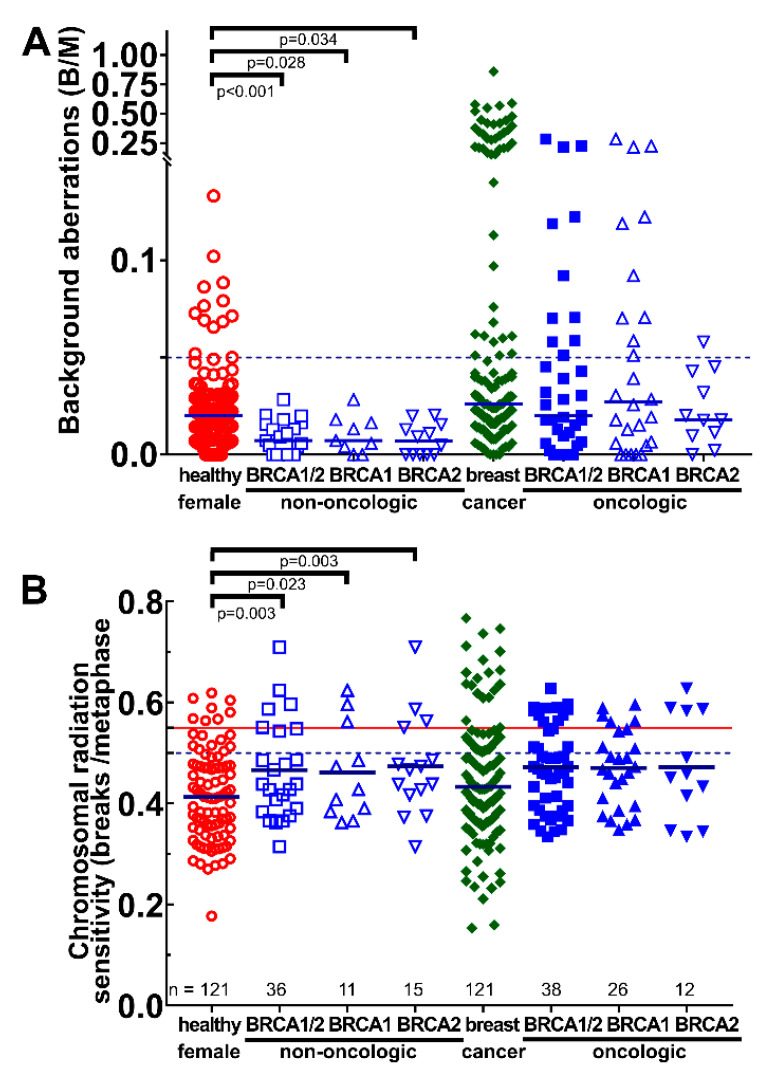
Chromosomal aberrations scored as breaks per metaphase in the non-oncologic and oncologic *BRCA1/2* cohorts. Healthy women were compared with all non-oncologic *BRCA1/2* or *BRCA1* or *BRCA2* subjects, and breast cancer patients were compared with oncologic *BRCA1/2* or *BRCA1* or *BRCA2* patients. (**A**) Background aberrations for breaks per metaphase and (**B**) chromosomal radiation sensitivity scored as breaks per metaphase after ex vivo irradiation with 2 Gy ionizing radiation and background correction. Horizontal lines depict 0.05 (**A**), and 0.5 and 0.55 (**B**) B/M cut-off values. B/M = Breaks per metaphase.

Therefore, next, the non-oncologic *BRCA1/2* were compared with the healthy female individuals and the oncologic *BRCA1/2* were compared with the patients with breast cancer. These two groups may be better controls because of their cancer and possible mutation status. Non-oncologic individuals with *BRCA1/2* had a clearly lower background B/M compared to the healthy female individuals (*p* < 0.034). Yet, there was no difference between breast cancer patients and oncologic *BRCA1/2*. What is strikingly very interesting is that the non-oncologic *BRCA1/2* have a significantly lower background of B/M compared to the oncologic *BRCA1/2* (*p* < 0.001) (Figure 3A). Similarly, in the ex vivo-irradiated lymphocytes, non-oncologic *BRCA1/2* subjects had a distinct increased B/M compared to healthy individuals (*p* < 0.023). Again, there were no differences between oncologic *BRCA1/2* and breast cancer patients (Figure 3B).

### 3.5. Radiation Sensitivity with Location or Type of BRCA1/2 Mutations

**Figure 4:**
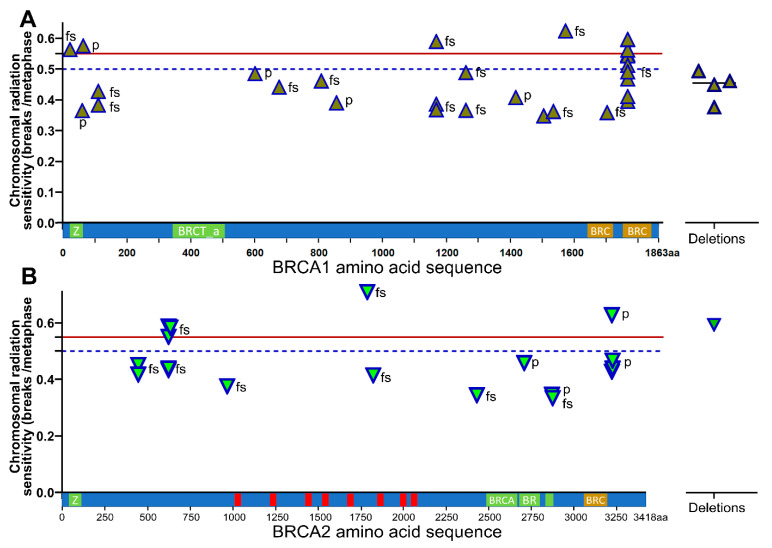
Chromosomal radiation sensitivity depending on the location of pathologic variants in the amino acid sequence of the *BRCA1* and *BRCA2* proteins. (**A**) Location of pathological variants in relation to radiosensitivity in the *BRCA1* protein; (**B**) Location of pathological variants in relation to radiosensitivity in the *BRCA2* protein. fs = Frame shift mutation; *p* = Point mutation.

An important question was whether a specific mutation or a specific location of the mutation particularly increases chromosomal radiosensitivity. In the *BRCA1* gene, most of the mutations were unique. We had two patients with mutations at positions p.111 (0.406 B/M) and p.1253 (0.427 B/M) and three mutations at position p.1161 (0.448 B/M). The only location of a mutation with a larger number of patients was p.1756 (p.Gln1756Pro fs*74) with ten patients. These ten patients had a significantly higher mean chromosomal radiosensitivity of 0.512 B/M compared to all others with a mean of 0.442 B/M (*p* = 0.027). Six out of these ten patients had B/M values higher than 0.5. The four patients with deletions in the *BRCA1* gene had no increased chromosomal radiosensitivity. Four other patients had values above 0.55 B/M. Three of them had mutations that occurred only once (p.Glu23Valfs*17; p.Cys64Tyr; Tyr1563Stop). One mutation was present three times with one B/M value above 0.55, and the two other B/M values were average (<0.4) (Glu1161Phefs*3) (Figure 4A).

In the *BRCA2* gene, two mutations each were at sites p.433 (0.434 B/M), aa3128 (0.452 B/M) and p.3124 (0.527 B/M). One patient in the latter group had a higher B/M value than 0.55. Four mutations were at p.605 with a high average value of 0.502 B/M, of which two patients again had a higher B/M value than 0.55. Only one patient had a deletion in the *BRCA2* gene, but with a high chromosomal radiosensitivity of 0.589 B/M (Figure 4B).

**Figure 5:**
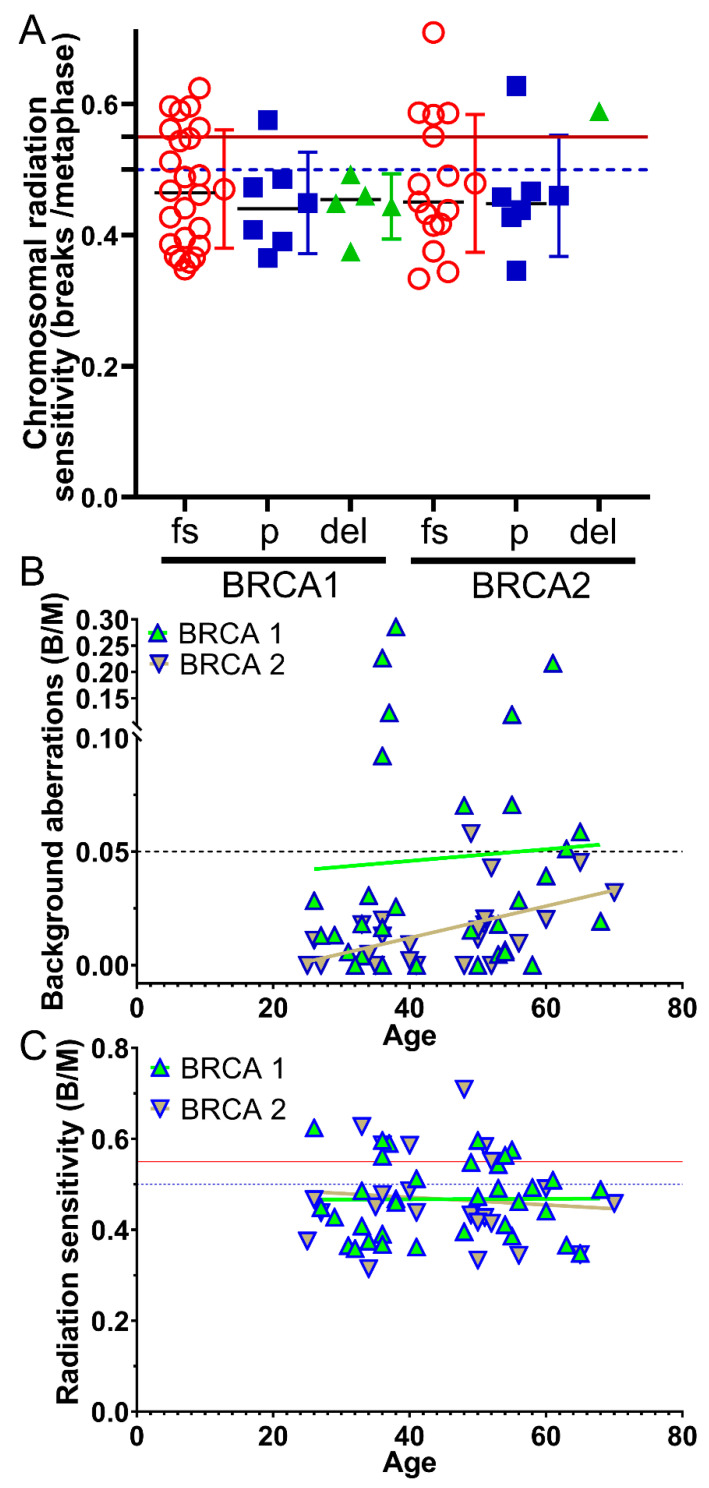
Dependence of type of mutation and age from radiation sensitivity. (**A**) Radiation sensitivity depending on the type of mutation in the *BRCA1* and *BRCA2* gene. fs = Frameshift mutation; *p* = Point mutation; del = Deletion. (**B**) Age-related dependence of background aberrations. (**C**) Age-related dependence of radiation sensitivity.

We also investigated whether a particular type of mutation (frameshift mutation, point mutation, deletion) is associated with high chromosomal radiosensitivity. Neither in the *BRCA1* nor in the *BRCA2* gene was a significantly increased chromosomal radiosensitivity linked to a specific mutation type (Figure 5A).

Lastly, we studied whether there is an association of radiation sensitivity with age in *BRCA1/2*-mutated patients. The *BRCA1* cohort was 45.1 years old (range 26–68) and the *BRCA2* cohort was 46.6 (25–70) years old. Background values for *BRCA1* were not associated with age (r^2^ = 0.044, *p* = 0.805), and only *BRCA2* showed a slight increase with age (r^2^ = 0.534, *p* = 0.009) (Figure 5B), while radiation sensitivity did not change with age for *BRCA1* (r^2^ = 0.008, *p* = 0.963) and *BRCA2* (r^2^ = −0.074, *p* = 0.736) (Figure 5C).

## 4. Discussion

In this study, we found that overall *BRCA1/2* variants do not confer clearly increased chromosomal radiosensitivity. However, some germline mutations in *BRCA1* and *BRCA2* lead to increased chromosomal radiosensitivity in the cohort. Individual variants may result in increased radiosensitivity. However, this would need to be confirmed in a study with a larger number of patients. It is difficult to obtain appropriate controls for this cohort. Therefore, we used different control cohorts for comparison. None of the controls have a known mutation status for *BRCA1/2* or other variants. This is because the cohorts were established historically, and healthy and tumor patients were not tested and therefore did not know their mutation status. Therefore, we can only estimate the mutation burden in the control cohorts. However, given that the prevalence of *BRCA1/2* mutations is estimated to be 1:400–1:500 in the general population [32,33] and 1:30–1:40 in the average breast cancer patient [34], it can be concluded that the vast majority of these patients are *BRCA1/2*-mutation-negative. Looking at the main breast cancer predisposition genes, 1.6% of the control group and 5.0% of the breast cancer cohort would have a pathologic mutation in a breast cancer gene. This is without knowing the overall effect of these genes on radiation sensitivity [4]. Pathologic variants in colorectal cancer predisposition genes are estimated to be present in 5–16% of colorectal tumors [35].

Our findings confirm the few available data samples on the irradiation of patients with germline *BRCA1/2* mutations. However, it has to be noted that these studies used slightly different techniques of analysis after irradiation than in our work [36,37,38,39]. Patients with somatic *BRCA1/2* mutations were also found to have above-average radiosensitivity in another retrospective analysis of medical records [40]. In our study, chromosomal radiosensitivity varied greatly from average chromosomal radiosensitivity to very high chromosomal radiosensitivity. Although no correlation with the type of mutation (e.g., frameshift, point mutation, deletion) could be found, there was a wide spectrum of radiosensitivity depending on the specific *BRCA1/2* mutation. In the *BRCA1* protein, the mutation p.Gln1756Pro fs*74 was associated with a distinct increased chromosomal radiosensitivity. However, even with this common mutation, affected patients showed a wide variety from moderate to high radiation sensitivity. There were four other single mutations in the *BRCA1* and *BRCA2* genes that were highly sensitive to radiation, but they were only present in a single case. The patient with the p.Glu1734Glyfs*9 mutation in the *BRCA2* gene encountered extreme high chromosomal radiosensitivity; yet, this mutation was also present in a single case. Because of this range of variation, other genetic and non-genetic factors could play a role in radiosensitive patients too, including single-nucleotide polymorphisms or mutations in other relevant genes as have been studied in non-*BRCA1/2*-mutated breast cancer patients [6,41,42].

DNA double-strand breaks, which are the most significant type of DNA damage induced by radiotherapy, are repaired via two distinct repair pathways: the non-homologous end joining (NHEJ) pathway and the HR pathway. Activation of either pathway also depends on the cell cycle phase. NHEJ is dominant in the G0/G1 phase while HR is dominant in the mid-S and mid-G2 phase. Both *BRCA1* and *BRCA2* play a crucial role in the repair of DNA DSBs [40]. If these DNA double-strand breaks are not repaired or are repaired incorrectly, chromosomal aberrations and mutations occur, which can be analyzed with the ex vivo three-color FiSH assay. Elevated mutation levels in the FiSH assay indicate disruptions in DNA double-strand break repair, signal transduction, cell cycle regulation and cell death control [23,43].

An important advantage of the three-color FiSH assay is its late endpoint, which makes it particularly suitable for determining individual radiation sensitivity. Another advantage is that with only the three colors and the chromosomes 1, 2 and 4, 22% of the total DNA can be screened for aberrations. In addition, the three colors are very easy and intuitive to recognize, making interpretation easier and more reliable [25,44]. After ex vivo irradiation, lymphocytes in G0 must undergo DNA repair and go through the entire cell cycle with all its checkpoints. Although the FiSH has great advantages for radiation sensitivity determination, individual radiation sensitivity is a complex construct that cannot always be addressed by a single test. However, the FiSH assay has been shown to predict radiosensitivity in several previous publications [43,45]. Individual radiosensitivity can be used to predict the risk of undesired side-effects of radiation therapy like fibrosis, skin toxicity or fatigue [19,46,47,48]. In addition, radiation sensitivity is genetically determined, so that the radiation sensitivity of lymphocytes can be extrapolated to all body cells and, in principle, to tumor cells.In a previously published paper, chromosomal radiosensitivity was increased by age; however, this finding was not significant, due to individual variability [21]. Here, among the *BRCA1/2* patients, no increase in radiation sensitivity with age was observed. However, non-oncologic *BRCA1/2* subjects had significantly fewer background aberrations compared with healthy controls and compared with oncologic *BRCA2*. Non-oncologic *BRCA1* (37.5 years) and *BRCA2* (38.8 years) were about 10 years younger than oncologic *BRCA1* (48.8 years) and *BRCA2* (51.0 years) and healthy females (49.5 years). The chromosomal background aberration increases with age [49] and this certainly explains most of this difference.

Chromosomal aberrations in the ex vivo-irradiated blood of non-oncological *BRCA1/2* patients were also significantly higher than in the healthy female control group. This is probably the most comparable group, as both represent non-oncologic subjects who once had *BRCA1/2* variants and once were very unlikely to have pathologic *BRCA1/2* variants in the healthy group. This clearly suggests that *BRCA1/2* contributes to an increase in radiation sensitivity, albeit to a limited extent. In oncologic *BRCA1/2* patients, B/M are similarly elevated compared with breast cancer patients, but without statistical significance. The control group of breast cancer patients certainly contains an unknown number of genetic variants of cancer genes and, therefore, probably, no such good separation is possible as in the healthy and the non-oncological *BRCA1/2* cohort. We emphasize the importance of measuring radiation sensitivity in individual patients suspected of having increased radiation sensitivity.

In such studies, it is important to have a large, controlled cohort to work with. In this study, there were 211 healthy individuals and 379 patients with rectal cancer as well as 147 patients with breast cancer. The majority of the patients were male. This is probably due to higher background levels in patients with rectal cancer because of their less balanced diet, smoking or other risk factors [50]. However, there were no sex-related differences in inducing chromosomal aberrations by ionizing radiation. Thus, males and females can be used equally as control groups. It is also necessary to determine the cut-off values for increased sensitivity to radiation from the control groups. The threshold for increased radiation sensitivity was set at 0.5 B/M [51]. This is the mean of a Gaussian distribution of a healthy cohort plus three times the standard deviation. However, recognizing that empirically derived therapy doses tend to be geared toward the more radiation-sensitive patients, the threshold above which we recommend dose reduction for patients has been set at 0.55 B/M. Establishing limits for radiation sensitivity is generally difficult. The risk of adverse effects beyond this threshold is still low and increases slowly with dose. Side-effects may not appear for years and are sometimes difficult to objectify. A very important factor in dose reductions is that recurrences do not increase, which would clearly indicate a false limit or dose reduction.

In patients with *BRCA1/2* mutations, reports on side-effects from irradiation are relatively rare and primarily retrospective [52]. In most studies, there were no increased late tissue side-effects in the *BRCA1/2*-mutated vs. non-mutated cohorts [53,54,55,56]. One explanation could be haploinsufficiency, which means that *BRCA1/2*-mutated patients only have a germline mutation in one allele with a second functional allele [52,57]. This is contradicted by the mutation analysis of our cohort of *BRCA1/2* mutation carriers, which clearly reveals a higher incidence of aberrations. Similarly, more chromatid breaks occurred in lymphocytes of heterozygous *BRCA1*-mutated female patients [58]. This could potentially go along with an increase in tissue damage. However, radiation after breast cancer in general has very little toxicity and is a well-tolerated standard therapy [59]. Therefore, the risk of undesirable side-effects is still low in patients with an increased sensitivity to radiation. For instance, only one patient from 170 accelerated partial breast irradiation patients in the ABPI2 study had a grade 2 pneumonitis [60]. Chromosomal radiosensitivity analysis in this patient showed a significantly increased chromosomal radiosensitivity of 0.79 B/M, presumably due to vitiligo. In another case, a 6-year-old boy with Phelan–McDermid syndrome and an atypical teratoid/rhabdoid tumor (AT/RT), who was measured to have a markedly elevated radiation sensitivity of 0.74 B/M, showed no adverse effects of therapy four years after treatment with a significant dose reduction from 54 Gy to 31 Gy [61]. A case of a three-year-old girl with the same genetic disease and tumor was reported in the literature. She received no dose adjustment and suffers from severe treatment sequelae [62]. This clearly demonstrates the relationship between significantly increased radiation sensitivity and increased risk of side-effects from therapy.

These representative cases illustrate that the risk of side-effects increases dramatically when there is significantly increased radiosensitivity, even with a very low toxic regimen. Another aspect is that when patients are treated with more toxic regimens, such as in head and neck cancer [63], and there is a *BRCA1/2* variant with increased radiosensitivity, the risk of adverse therapeutic effects would increase significantly. Consequently, the identification of radiosensitive patients is of interest, especially with regard to specific pathologic variants or specific areas in the associated proteins that are linked to higher radiosensitivity. Such deeper knowledge would avoid the need for time-consuming radiosensitivity testing.

## 5. Conclusions

Patients with germline *BRCA1/2* mutations have very different levels of chromosomal radiosensitivity, ranging from average to clearly elevated chromosomal radiosensitivity in an ex vivo FiSH assay. At present, radiosensitivity cannot be predicted by general types of mutations, e.g., point mutation, frameshift mutation or deletion. However, it appears that certain mutations in *BRCA1* and *BRCA2* are associated with higher rates of chromosome breaks in the ex vivo FiSH assay. With conservative fractionated breast radiotherapy, the risk of adverse events in these patients appears to be limited [64]. However, there may be an increased risk of using a regimen with higher toxicity that could lead to increased side-effects in patients with certain *BRCA1/2* mutations. This study justifies further research on radiosensitivity in patients with mutations in *BRCA1/2* and also other genes involved in DNA repair, requiring larger numbers of patients studied.

## Figures and Tables

**Table 1:** Characteristics of patients with *BRCA1/2* pathologic variants.

			*BRCA1/2* *n* = 64 (%)	*BRCA1* *n* = 37 (%)	*BRCA2* *n* = 27 (%)
	Mean age (range, years)		45.7 (25–70)	45.1 (26–68)	46.6 (25–70)
	Mean height (m)		1.65	1.66	1.65
	Mean weight (kg)		70.7	71.9	69.3
	Mean BMI (kg/m^2^)		25.7	26.0	25.3
	Premenopausal		32 (50.0)	19 (51.4)	13 (48.1)
	Postmenopausal		30 (46.9)	16 (43.2)	14 (40.7)
	Not known		2 (3.1)	2 (5.4)	0
	No breast cancer		21 (32.8)	9 (24.3)	12 (44.4)
	Breast cancer		38 (59.4)	26 (70.3)	12 (44.4)
	Breast cancer status not known		5 (7.8)	2 (5.4)	3 (11.1)
Tumor stage		Tis	2 (3.1)	2 (7.7)	0
	T1		19 (29.7)	9 (34.6)	10 (83.3)
	T2		12 (18.8)	10 (38.5)	2 (16.7)
	T3		2 (3.1)	2 (7.7)	0
	T4		0	0	0
	Not known		3 (4.7)	3 (11.5)	0
Regional lymph nodes		N0	26 (40.6)	16 (61.5)	10 (83.3)
	N+		9 (14.1)	7 (27)	2 (16.7)
	Not known		3 (4.7)	3 (11.5)	0
Distant metastasis		M0	33 (51.6)	21 (80.8)	12 (100)
	M1		0	0	0
	Mx		5 (7.8)	5 (19.2)	0
Receptors	ER or PR positive		10 (15.6)	8 (30.8)	2 (16.7)
	Triple negative		19 (29.7)	11 (42.3)	8 (66.7)
	HER2/neu positive		7 (10.9)	5 (19.2)	2 (16.7)
	Not known		2 (3.1)	2 (7.7)	0
Grading		G1	1 (1.6)	1 (3.8)	0
	G2		6 (9.4)	4 (15.4)	2 (16.7)
	G3		27 (42.2)	17 (65.4)	10 (83.3)
	Not known		4 (6.3)	4 (15.4)	0
	Mean B/M values		0.47	0.46	0.48

B/M = Breaks per metaphase; Tis = Tumor in situ.

**Table 2:** Proportion of individuals in each cohort with chromosomal aberrations above specified thresholds.

				Healthy		Rectal Cancer		Breast
	Threshold	*BRCA1*	*BRCA2*	Male	Female	Male	Female	Cancer
Background (%)	≥0.05	27.8	8.7	6.7	9.9	36.7	25.2	30.6
Radiation sensitives (%)	≥0.5	30.6	30.4	18.9	14.9	23.9	27.3	29.9
	≥0.55	19.4	30.4	8.9	5.8	17.6	15.2	12.9
	≥0.6	2.8	8.7	3.3	1.7	11.9	10.6.	11.6

**Table 3:** B/M values in all analyzed cohorts. Cohorts of healthy individuals and patients with rectal cancer and breast cancer have unknown *BRCA1/2* mutation status.

	Mean B/M Value (2 Gy)	*p*-Value	95% Confidence Interval
Patients with a *BRCA1/2* mutation (*n* = 64)	0.468	---	0.445 to 0.491
Healthy individuals (*n* = 215)	0.411	<0.001	0.399 to 0.423
Patients with rectal cancer (*n* = 385)	0.439	0.039	0.420 to 0.459
Patients with breast cancer (*n* = 147)	0.447	0.235	0.426 to 0.468

## Data Availability

All data are available in the main text. Further data can be obtained from the corresponding author on request.

## Abbreviations

ACMG	American College of Medical Genetics and Genomics
AWMF	Arbeitsgemeinschaft der Wissenschaftlichen Medizinischen Fachgesellschaften e.V.
B/M	Breaks per metaphase
*BRCA1/2*	BReast CAncer 1/2
DAPI	4′,6-diamidin-2-phenylindol
FiSH	Fluorescence in situ hybridization
HR	Homologous recombination
IR	Ionizing radiation;
NHEJ	Non homologous end joining
PARP	poly-ADP-ribose polymerase
RPMI	Roswell Park Memorial Institute Medium

## References

[1] Arnold M., Morgan E., Rumgay H., Mafra A., Singh D., Laversanne M., Vignat J., Gralow J.R., Cardoso F., Siesling S. (2022). Current and future burden of breast cancer: Global statistics for 2020 and 2040. Breast.

[2] Sung H., Ferlay J., Siegel R.L., Laversanne M., Soerjomataram I., Jemal A., Bray F. (2021). Global Cancer Statistics 2020: GLOBOCAN Estimates of Incidence and Mortality Worldwide for 36 Cancers in 185 Countries. CA Cancer J. Clin..

[3] Howlader N., Noone A.M., Miller D., Krapcho M., Brest A., Yu M., Ruhl J., Tatalovich Z., Mariotto A., Lewis D.R. SEER Cancer Statistics Review, 1975–2017; Based on November 2019 SEER Data Submission, Posted to the SEER Web Site; National Cancer Institute: Bethesda, MD, USA, 2020. https://seer.cancer.gov/csr/1975_2017.

[4] Hu C., Hart S.N., Gnanaolivu R., Huang H., Lee K.Y., Na J., Gao C., Lilyquist J., Yadav S., Boddicker N.J. (2021). A Population-Based Study of Genes Previously Implicated in Breast Cancer. N. Engl. J. Med..

[5] Brewer H.R., Jones M.E., Schoemaker M.J., Ashworth A., Swerdlow A.J. (2017). Family history and risk of breast cancer: An analysis accounting for family structure. Breast Cancer Res. Treat..

[6] Dorling L., Carvalho S., Allen J., Gonzalez-Neira A., Luccarini C., Wahlstrom C., Pooley K.A., Parsons M.T., Fortuno C., Wang Q. (2021). Breast Cancer Risk Genes-Association Analysis in More than 113,000 Women. N. Engl. J. Med..

[7] Chen S., Parmigiani G. (2007). Meta-analysis of BRCA1 and BRCA2 penetrance. J. Clin. Oncol..

[8] Kuchenbaecker K.B., Hopper J.L., Barnes D.R., Phillips K.A., Mooij T.M., Roos-Blom M.J., Jervis S., van Leeuwen F.E., Milne R.L., Andrieu N. (2017). Risks of Breast, Ovarian, and Contralateral Breast Cancer for BRCA1 and BRCA2 Mutation Carriers. JAMA.

[9] Li S., Silvestri V., Leslie G., Rebbeck T.R., Neuhausen S.L., Hopper J.L., Nielsen H.R., Lee A., Yang X., McGuffog L. (2022). Cancer Risks Associated With BRCA1 and BRCA2 Pathogenic Variants. J. Clin. Oncol..

[10] El-Nachef L., Al-Choboq J., Restier-Verlet J., Granzotto A., Berthel E., Sonzogni L., Ferlazzo M.L., Bouchet A., Leblond P., Combemale P. (2021). Human Radiosensitivity and Radiosusceptibility: What Are the Differences?. Int. J. Mol. Sci..

[11] Yoshida K., Miki Y. (2004). Role of BRCA1/2 and BRCA1/2 as regulators of DNA repair, transcription, and cell cycle in response to DNA damage. Cancer Sci..

[12] Gorodetska I., Kozeretska I., Dubrovska A. (2019). BRCA Genes: The Role in Genome Stability, Cancer Stemness and Therapy Resistance. J. Cancer.

[13] Wöckel A., Festl J., Stüber T., Brust K., Stangl S., Heuschmann P.U., Albert U.S., Budach W., Follmann M., Janni W. (2018). Interdisciplinary Screening, Diagnosis, Therapy and Follow-up of Breast Cancer. Guideline of the DGGG and the DKG (S3-Level, AWMF Registry Number 032/045OL, December 2017)-Part 1 with Recommendations for the Screening, Diagnosis and Therapy of Breast Cancer. Geburtshilfe Frauenheilkd..

[14] Fahrig A., Koch T., Lenhart M., Rieckmann P., Fietkau R., Distel L., Schuster B. (2018). Lethal outcome after pelvic salvage radiotherapy in a patient with prostate cancer due to increased radiosensitivity: Case report and literature review. Strahlenther. Onkol..

[15] Zhou Y., Yan T., Zhou X., Cao P., Luo C., Zhou L., Xu Y., Liu Y., Xue J., Wang J. (2020). Acute severe radiation pneumonitis among non-small cell lung cancer (NSCLC) patients with moderate pulmonary dysfunction receiving definitive concurrent chemoradiotherapy: Impact of pre-treatment pulmonary function parameters. Strahlenther. Onkol..

[16] Klement R.J., Schäfer G., Sweeney R.A. (2019). A fatal case of Fournier’s gangrene during neoadjuvant radiotherapy for rectal cancer. Strahlenther. Onkol..

[17] Scheckenbach K., Wagenmann M., Freund M., Schipper J., Hanenberg H. (2012). Squamous cell carcinomas of the head and neck in Fanconi anemia: Risk, prevention, therapy, and the need for guidelines. Klin. Padiatr..

[18] Taylor A.M., Harnden D.G., Arlett C.F., Harcourt S.A., Lehmann A.R., Stevens S., Bridges B.A. (1975). Ataxia telangiectasia: A human mutation with abnormal radiation sensitivity. Nature.

[19] Hoeller U., Borgmann K., Bonacker M., Kuhlmey A., Bajrovic A., Jung H., Alberti W., Dikomey E. (2003). Individual radiosensitivity measured with lymphocytes may be used to predict the risk of fibrosis after radiotherapy for breast cancer. Radiother. Oncol..

[20] Auer J., Keller U., Schmidt M., Ott O., Fietkau R., Distel L.V. (2014). Individual radiosensitivity in a breast cancer collective is changed with the patients’ age. Radiol. Oncol..

[21] Schuster B., Ellmann A., Mayo T., Auer J., Haas M., Hecht M., Fietkau R., Distel L.V. (2018). Rate of individuals with clearly increased radiosensitivity rise with age both in healthy individuals and in cancer patients. BMC Geriatr..

[22] Schuster B., Hecht M., Schmidt M., Haderlein M., Jost T., Buttner-Herold M., Weber K., Denz A., Grutzmann R., Hartmann A. (2021). Influence of Gender on Radiosensitivity during Radiochemotherapy of Advanced Rectal Cancer. Cancers.

[23] Stritzelberger J., Lainer J., Gollwitzer S., Graf W., Jost T., Lang J.D., Mueller T.M., Schwab S., Fietkau R., Hamer H.M. (2020). Ex vivo radiosensitivity is increased in non-cancer patients taking valproate. BMC Neurol..

[24] Dunst J., Gebhart E., Neubauer S. (1995). Can an extremely elevated radiosensitivity in patients be recognized by the in-vitro testing of lymphocytes?. Strahlenther. Onkol..

[25] Neubauer S., Dunst J., Gebhart E. (1997). The impact of complex chromosomal rearrangements on the detection of radiosensitivity in cancer patients. Radiother. Oncol..

[26] Keller U., Kuechler A., Liehr T., Muller E., Grabenbauer G., Sauer R., Distel L. (2004). Impact of Various Parameters in Detecting Chromosomal Aberrations by FISH to Describe Radiosensitivity. Strahlenther. Onkol..

[27] Stritzelberger J., Distel L., Buslei R., Fietkau R., Putz F. (2018). Acquired temozolomide resistance in human glioblastoma cell line U251 is caused by mismatch repair deficiency and can be overcome by lomustine. Clin. Transl. Oncol..

[28] Savage J.R., Simpson P. (1994). On the scoring of FISH-"painted" chromosome-type exchange aberrations. Mutat. Res..

[29] Hecht M., Zimmer L., Loquai C., Weishaupt C., Gutzmer R., Schuster B., Gleisner S., Schulze B., Goldinger S.M., Berking C. (2015). Radiosensitization by BRAF inhibitor therapy-mechanism and frequency of toxicity in melanoma patients. Ann. Oncol..

[30] Weigert V., Jost T., Hecht M., Knippertz I., Heinzerling L., Fietkau R., Distel L.V. (2020). PARP inhibitors combined with ionizing radiation induce different effects in melanoma cells and healthy fibroblasts. BMC Cancer.

[31] Dobler C., Jost T., Hecht M., Fietkau R., Distel L. (2020). Senescence Induction by Combined Ionizing Radiation and DNA Damage Response Inhibitors in Head and Neck Squamous Cell Carcinoma Cells. Cells.

[32] Anglian Breast Cancer Study Group (2000). Prevalence and penetrance of BRCA1 and BRCA2 mutations in a population-based series of breast cancer cases. Anglian Breast Cancer Study Group. Br. J. Cancer.

[33] Whittemore A.S., Gong G., John E.M., McGuire V., Li F.P., Ostrow K.L., Dicioccio R., Felberg A., West D.W. (2004). Prevalence of BRCA1 mutation carriers among U.S. non-Hispanic Whites. Cancer Epidemiol. Biomark. Prev..

[34] Armstrong N., Ryder S., Forbes C., Ross J., Quek R.G. (2019). A systematic review of the international prevalence of BRCA mutation in breast cancer. Clin. Epidemiol..

[35] Rebuzzi F., Ulivi P., Tedaldi G. (2023). Genetic Predisposition to Colorectal Cancer: How Many and Which Genes to Test?. Int. J. Mol. Sci..

[36] Sadeghi F., Asgari M., Matloubi M., Ranjbar M., Karkhaneh Yousefi N., Azari T., Zaki-Dizaji M. (2020). Molecular contribution of BRCA1 and BRCA2 to genome instability in breast cancer patients: Review of radiosensitivity assays. Biol. Proced. Online.

[37] Baert A., Depuydt J., Van Maerken T., Poppe B., Malfait F., Storm K., van den Ende J., Van Damme T., De Nobele S., Perletti G. (2016). Increased chromosomal radiosensitivity in asymptomatic carriers of a heterozygous BRCA1 mutation. Breast Cancer Res..

[38] Ernestos B., Nikolaos P., Koulis G., Eleni R., Konstantinos B., Alexandra G., Michael K. (2010). Increased chromosomal radiosensitivity in women carrying BRCA1/BRCA2 mutations assessed with the G2 assay. Int. J. Radiat. Oncol. Biol. Phys..

[39] Baeyens A., Thierens H., Claes K., Poppe B., Messiaen L., De Ridder L., Vral A. (2002). Chromosomal radiosensitivity in breast cancer patients with a known or putative genetic predisposition. Br. J. Cancer.

[40] Kim K.H., Kim H.S., Kim S.S., Shim H.S., Yang A.J., Lee J.J.B., Yoon H.I., Ahn J.B., Chang J.S. (2022). Increased Radiosensitivity of Solid Tumors Harboring ATM and BRCA1/2 Mutations. Cancer Res. Treat..

[41] Barnett G.C., Coles C.E., Elliott R.M., Baynes C., Luccarini C., Conroy D., Wilkinson J.S., Tyrer J., Misra V., Platte R. (2012). Independent validation of genes and polymorphisms reported to be associated with radiation toxicity: A prospective analysis study. Lancet Oncol..

[42] Barnett G.C., Thompson D., Fachal L., Kerns S., Talbot C., Elliott R.M., Dorling L., Coles C.E., Dearnaley D.P., Rosenstein B.S. (2014). A genome wide association study (GWAS) providing evidence of an association between common genetic variants and late radiotherapy toxicity. Radiother. Oncol..

[43] Beaton L.A., Marro L., Samiee S., Malone S., Grimes S., Malone K., Wilkins R.C. (2013). Investigating chromosome damage using fluorescent in situ hybridization to identify biomarkers of radiosensitivity in prostate cancer patients. Int. J. Radiat. Biol..

[44] Dunst J., Neubauer S., Becker A., Gebhart E. (1998). Chromosomal in-vitro radiosensitivity of lymphocytes in radiotherapy patients and AT-homozygotes. Strahlenther. Onkol..

[45] Mayo T., Haderlein M., Schuster B., Wiesmüller A., Hummel C., Bachl M., Schmidt M., Fietkau R., Distel L. (2019). Is in vivo and ex vivo irradiation equally reliable for individual Radiosensitivity testing by three colour fluorescence in situ hybridization?. Radiat. Oncol..

[46] Borgmann K., Hoeller U., Nowack S., Bernhard M., Roper B., Brackrock S., Petersen C., Szymczak S., Ziegler A., Feyer P. (2008). Individual radiosensitivity measured with lymphocytes may predict the risk of acute reaction after radiotherapy. Int. J. Radiat. Oncol. Biol. Phys..

[47] Liu L., Yang Y., Guo Q., Ren B., Peng Q., Zou L., Zhu Y., Tian Y. (2020). Comparing hypofractionated to conventional fractionated radiotherapy in postmastectomy breast cancer: A meta-analysis and systematic review. Radiat. Oncol..

[48] Hauth F., De-Colle C., Weidner N., Heinrich V., Zips D., Gani C. (2021). Quality of life and fatigue before and after radiotherapy in breast cancer patients. Strahlenther. Onkol..

[49] Bolognesi C., Abbondandolo A., Barale R., Casalone R., Dalpra L., De Ferrari M., Degrassi F., Forni A., Lamberti L., Lando C. (1997). Age-related increase of baseline frequencies of sister chromatid exchanges, chromosome aberrations, and micronuclei in human lymphocytes. Cancer Epidemiol. Biomark. Prev..

[50] Lewandowska A., Rudzki G., Lewandowski T., Stryjkowska-Gora A., Rudzki S. (2022). Title: Risk Factors for the Diagnosis of Colorectal Cancer. Cancer Control. J. Moffitt Cancer Cent..

[51] Keller U., Grabenbauer G., Kuechler A., Sprung C., Müller E., Sauer R., Distel L. (2005). Cytogenetic instability in young patients with multiple primary cancers. Cancer Genet. Cytogenet..

[52] Lazzari G., Buono G., Zannino B., Silvano G. (2021). Breast Cancer Adjuvant Radiotherapy in BRCA1/2, TP53, ATM Genes Mutations: Are There Solved Issues?. Breast Cancer (Dove Med. Press).

[53] Huszno J., Budryk M., Kolosza Z., Nowara E. (2015). The risk factors of toxicity during chemotherapy and radiotherapy in breast cancer patients according to the presence of BRCA gene mutation. Contemp. Oncol. (Pozn.).

[54] Park H., Choi D.H., Noh J.M., Huh S.J., Park W., Nam S.J., Lee J.E. (2014). Acute skin toxicity in Korean breast cancer patients carrying BRCA mutations. Int. J. Radiat. Biol..

[55] Pierce L.J., Levin A.M., Rebbeck T.R., Ben-David M.A., Friedman E., Solin L.J., Harris E.E., Gaffney D.K., Haffty B.G., Dawson L.A. (2006). Ten-year multi-institutional results of breast-conserving surgery and radiotherapy in BRCA1/2-associated stage I/II breast cancer. J. Clin. Oncol..

[56] Shanley S., McReynolds K., Ardern-Jones A., Ahern R., Fernando I., Yarnold J., Evans G., Eccles D., Hodgson S., Ashley S. (2006). Late toxicity is not increased in BRCA1/BRCA2 mutation carriers undergoing breast radiotherapy in the United Kingdom. Clin. Cancer Res..

[57] Elalaoui S.C., Laarabi F.Z., Afif L., Lyahyai J., Ratbi I., Jaouad I.C., Doubaj Y., Sahli M., Ouhenach M., Sefiani A. (2022). Mutational spectrum of BRCA1/2 genes in Moroccan patients with hereditary breast and/or ovarian cancer, and review of BRCA mutations in the MENA region. Breast Cancer Res. Treat..

[58] Febrer E., Mestres M., Caballín M.R., Barrios L., Ribas M., Gutiérrez-Enríquez S., Alonso C., Ramón y Cajal T., Francesc Barquinero J. (2008). Mitotic delay in lymphocytes from BRCA1 heterozygotes unable to reduce the radiation-induced chromosomal damage. DNA Repair. (Amst.).

[59] Wang K., Tepper J.E. (2021). Radiation therapy-associated toxicity: Etiology, management, and prevention. CA Cancer J. Clin..

[60] Ott O.J., Stillkrieg W., Lambrecht U., Sauer T.O., Schweizer C., Lamrani A., Strnad V., Hack C.C., Beckmann M.W., Uder M. (2022). External Beam Accelerated Partial Breast Irradiation in Early Breast Cancer and the Risk for Radiogenic Pneumonitis. Cancers.

[61] De Rubeis S., Siper P.M., Durkin A., Weissman J., Muratet F., Halpern D., Trelles M.D.P., Frank Y., Lozano R., Wang A.T. (2018). Delineation of the genetic and clinical spectrum of Phelan-McDermid syndrome caused by SHANK3 point mutations. Mol. Autism.

[62] Byers H.M., Adam M.P., LaCroix A., Leary S.E., Cole B., Dobyns W.B., Mefford H.C. (2017). Description of a new oncogenic mechanism for atypical teratoid rhabdoid tumors in patients with ring chromosome 22. Am. J. Med. Genet. A.

[63] Anderson G., Ebadi M., Vo K., Novak J., Govindarajan A., Amini A. (2021). An Updated Review on Head and Neck Cancer Treatment with Radiation Therapy. Cancers.

[64] Mangesius J., Minasch D., Fink K., Nevinny-Stickel M., Lukas P., Ganswindt U., Seppi T. (2022). Systematic risk analysis of radiation pneumonitis in breast cancer: Role of cotreatment with chemo-, endocrine, and targeted therapy. Strahlenther. Onkol..

